# Functional Imaging of Stimulus Convergence in Amygdalar Neurons during Pavlovian Fear Conditioning

**DOI:** 10.1371/journal.pone.0006156

**Published:** 2009-07-07

**Authors:** Sabiha K. Barot, Ain Chung, Jeansok J. Kim, Ilene L. Bernstein

**Affiliations:** 1 Program in Neurobiology & Behavior, University of Washington, Seattle, Washington, United States of America; 2 Department of Psychology, University of Washington, Seattle, Washington, United States of America; University of Groningen, Netherlands

## Abstract

**Background:**

Associative conditioning is a ubiquitous form of learning throughout the animal kingdom and fear conditioning is one of the most widely researched models for studying its neurobiological basis. Fear conditioning is also considered a model system for understanding phobias and anxiety disorders. A fundamental issue in fear conditioning regards the existence and location of neurons in the brain that receive convergent information about the conditioned stimulus (CS) and unconditioned stimulus (US) during the acquisition of conditioned fear memory. Convergent activation of neurons is generally viewed as a key event for fear learning, yet there has been almost no direct evidence of this critical event in the mammalian brain.

**Methodology/Principal Findings:**

Here, we used *A*rc cellular *c*ompartmental *a*nalysis of *t*emporal gene transcription by *f*luorescence *i*n *s*itu *h*ybridization (catFISH) to identify neurons activated during single trial contextual fear conditioning in rats. To conform to temporal requirements of catFISH analysis we used a novel delayed contextual fear conditioning protocol which yields significant single- trial fear conditioning with temporal parameters amenable to catFISH analysis. Analysis yielded clear evidence that a population of BLA neurons receives convergent CS and US information at the time of the learning, that this only occurs when the CS-US arrangement is supportive of the learning, and that this process requires *N*-methyl-D-aspartate receptor activation. In contrast, CS-US convergence was not observed in dorsal hippocampus.

**Conclusions/Significance:**

Based on the pattern of *Arc* activation seen in conditioning and control groups, we propose that a key requirement for CS-US convergence onto BLA neurons is the potentiation of US responding by prior exposure to a novel CS. Our results also support the view that contextual fear memories are encoded in the amygdala and that the role of dorsal hippocampus is to process and transmit contextual CS information.

## Introduction

Neurobiological models of basic associative conditioning propose that neurons critical to learning receive convergent information from pathways responsive to the CS and US, and that activity-dependent changes in these neurons encode the formation of the associative memory trace [1]–[3]. In mammalian fear conditioning, where an initially innocuous CS becomes capable of evoking conditioned fear responses (CRs) after contingent pairing with an aversive US [4], [5], long-lasting synaptic plasticity and learning-induced changes in cellular and molecular activity have been demonstrated in the BLA (basal and lateral nuclei of the amygdala), a brain region implicated in the encoding of fear memory [6]–[11]. However, the crucial evidence yet to be secured is whether a population of amygdalar neurons receives convergent information at the time of training, and if it does so only when the CS-US arrangement produces fear conditioning.

Using electrophysiological methods, several studies have shown learning-induced changes in amygdalar neurons following fear conditioning. For instance, tone-evoked potentials recorded in neurons of the lateral amygdala (LA) have been shown to increase after auditory fear conditioning [12], and the magnitude of long-term potentiation (LTP) is larger in the BLA of fear conditioned rats compared to those of control rats [13]–[15].

Fear conditioning and LTP have also been associated with increased induction of *Arc* (or *Arg3.1*), an immediate early gene expressed in glutamatergic neurons, in the BLA [16], [17]. The importance of *Arc* in the acquisition of conditioned fear is underscored by reports that viral-mediated overexpression of cAMP response element binding protein (CREB) in BLA enhances fear learning and increases the number of *Arc*+ neurons in the amygdala of wild type and CREB knockout mice [18]; and that knock-down of *Arc* mRNA in LA interferes with auditory fear conditioning in rats [16]. However, *Arc* assessment after CS-US pairing in these studies could not distinguish between CS responsive, US responsive, and both CS and US responsive neurons, so it remains unclear whether observed responses occurred in neuronal populations receiving convergent activations.

To our knowledge then, there is no definitive evidence that CS-US information converges on individual neurons in the amygdala at the time of fear conditioning. In the present study, we employed the functional imaging technique *Arc* catFISH to distinguish neuronal populations activated by a behavioral experience with the CS and US. CatFISH utilizes the dynamic compartmentalization of *Arc* mRNA as a time stamp for recent neuronal activity: following induction, *Arc* mRNA is confined to the nucleus for about 5 minutes, after which it moves to cytoplasm where it is completely restricted by ∼25–30 minutes [19], [20]. Thus by using the subcellular distribution of *Arc* mRNA, catFISH analysis can mark neuronal populations engaged by the CS, the US, and the pairing of the two stimuli during fear learning.

**Figure 1:**
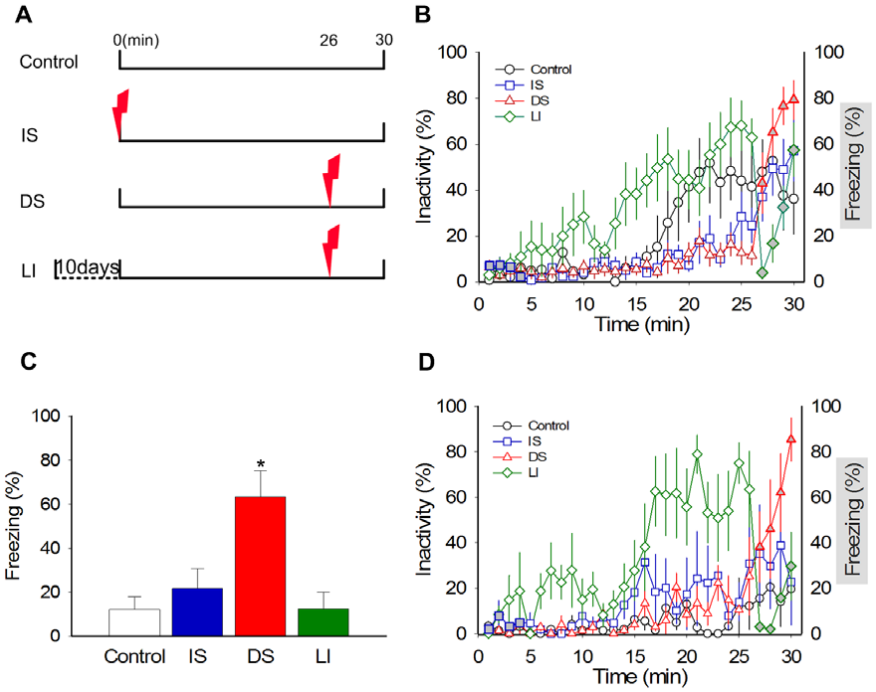
Single Trial Fear Conditioning. (A) A schematic timeline outlining presentation of the context CS and footshock US for Controls, Immediate Shock (IS), Delay Shock (DS), and Latent Inhibition (LI) groups during training. (B) Freezing behavior on training day. Animals introduced to the novel chamber initially explored the context and then became less active as the chamber became familiar. This was especially evident in the LI group. IS animals showed minimal freezing following footshock. In contrast, DS animals, and LI animals to a lesser extent, showed robust freezing following shock (*P* = .01). Grey-filled symbols represent the period when postshock freezing was assessed in DS, IS and LI animals. (C) Freezing behavior on retention test next day. Only DS animals showed significant freezing 24 hours after conditioning (*P* = .01). (D) Animals used for catFISH analysis showed similar trends of activity and freezing during training as behaviorally tested animals. These animals were sacrificed immediately after 30 minutes in the chamber. Data are represented as percent means±SEM.

However, because catFISH analysis requires that presentation of stimuli be separated by ∼25 min, a fear conditioning protocol had to be modified to make it amenable to this analysis (Figure 1A). An initial behavioral study indicated that contextual fear conditioning can occur in a single trial when introduction to a novel context CS is followed 26 min later by delivery of footshock US. Subsequently, catFISH analysis of sections from both the BLA (the putative site of fear conditioning) [6]–[11] and dorsal hippocampus (implicated in processing context-spatial information) [20]–[23], allowed us to determine whether neurons in these regions are dually activated by the CS and US during acquisition of conditioned fear.

## Results

### Single trial contextual fear conditioning

When placed in a novel chamber for 30 min, control rats exhibited several minutes of active exploration after which they became less active and, in some cases, went to sleep. The delayed shock (DS) rats behaved similarly to controls, but following footshock, they demonstrated robust freezing (an index of fear) during the last 4 min postshock period in the chamber (Figure 1B). In contrast, the immediate shock (IS) group, which experienced footshock instantly upon placement in the chamber [24], displayed virtually no postshock freezing (5.6±2.9%, for an equivalent 4 min postshock period) and behaved like the control group for the remaining time in the chamber. Latent inhibition (LI) rats experienced the same chamber-footshock interval as the DS rats, but because of substantial context pre-exposure, exhibited earlier and larger episodes of inactivity/sleep in the chamber than other groups preceding the footshock (group main effects: *F*_3,27_ = 5.578, *P* = .004; *P's*<.05 for LI vs.DS/IS, Tukey's *HSD*) and significantly less postshock freezing (42.5±13.8%) than DS animals (71.8+8.7%). Indeed, among animals that received footshock (i.e., DS, IS and LI groups), ANOVAs indicate no main effect of group during the first 4 minutes in the chamber (*F*_2,21_ = .092, *P* = .913) but a significant main effect of group during the last 4 (postshock) minutes in the chamber (*F*_2,21_ = 21.199, *P* = .000; *P's*<.05 for DS vs. IS & LI groups, Tukey's *HSD*).

The next day, when rats were placed back in the test chamber for 8 min to assess the retention of contextual fear memory, similar group effects were observed (Figure 1C). Only DS animals showed evidence of having acquired a conditioned fear response, as indicated by robust freezing; whereas control, IS and LI animals did not (group main effects: *F*_3, 27_ = 7.53, *P* = .001; *P's*<.05 for DS vs. all other groups, Tukey's *HSD*). These behavioral data indicate that significant contextual fear conditioning can be acquired using temporal parameters that are compatible with catFISH analysis. Specifically, animals can acquire long-term fear associations when a *novel* context is paired with shock 26 minutes later (DS group), but not when a *familiar* context is similarly paired with shock (LI group).

### *A*rc catFISH in the amygdala

Rats used for catFISH analysis underwent the same control, DS, IS, and LI training as above, except they were sacrificed promptly 30 min after introduction into the chamber. Once again, control and IS animals exhibited essentially no freezing, and DS animals displayed significantly more freezing than LI animals during the 4 minute post-shock period (Figure 1D).

**Figure 2:**
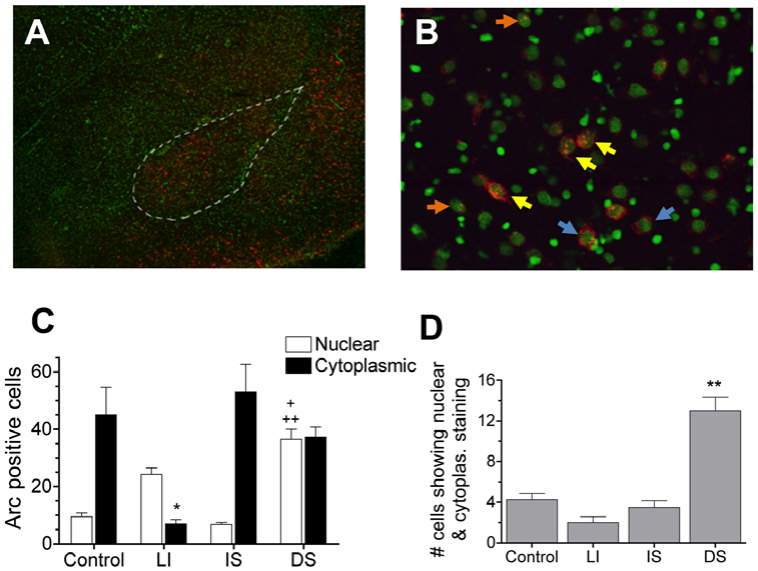
*Arc* induction in the BLA is sensitive to both the CS and US. (A) A representative image (4×) of the BLA displaying *Arc^+^* staining (in red, nuclei are counter-stained green) following DS fear conditioning. (B) A higher magnification image (4×) of the BLA showing cytoplasmic *Arc^+^* staining (blue arrows), nuclear *Arc* ^+^ staining (orange arrows), and both nuclear and cytoplasmic staining (yellow arrows). For DS animals, cytoplasmic staining corresponds to the context CS event and nuclear staining corresponds to the footshock US event. (C) Total number of cells in the BLA with *Arc^+^* cytoplasmic and nuclear staining across groups (n = 4 rats/group). (Note: for IS animals, cytoplasmic staining corresponds to both CS and US stimuli). The total number of neurons with *Arc* ^+^ cytoplasmic staining was significantly lower for context-familiar LI animals than for all other groups (*P<.01), indicating that *Arc* induction in BLA is sensitive to a novel CS. Moreover, groups receiving shock four minutes prior to sacrifice (DS and LI) show significant levels of nuclear staining compared to Controls, indicating that *Arc* is also sensitive to the US (++*P*<.005). Interestingly, DS animals showed a significantly greater number of neurons responding to the US than LI animals (+*P*<.01). (D) Number of neurons showing dual *Arc^+^* staining in both the nucleus and cytoplasm across groups. Only animals in the DS group showed a significant number of neurons responding to both the CS and US (**P<.001). Data are represented as means±SEM.

Introduction to a novel context (CS) and delivery of shock (US) were effective in promoting *Arc* mRNA expression in BLA neurons (Figure 2A–B). Because the central nucleus (CeA) showed negligible *Arc* ^+^ neurons following conditioning, analysis was confined to the BLA. The CS-induced *Arc* signal was always cytoplasmic since introduction to the test chamber occurred 30 min before sacrifice for all groups (Figure 2C). However the location of US-induced *Arc* signal varied between the IS and DS/LI groups since the former received a shock 30 minutes prior to sacrifice whereas the latter two received it only 4 minutes prior (no shock-induced *Arc* was seen in control animals since they did not receive the US). Moreover, the LI group showed little or no *Arc* signal to the chamber, indicating that *Arc* induction in BLA is sensitive to context novelty (group main effects: *F*_3, 12_ = 7.99, *P* = .003; *P's*<.01 for LI vs. all other groups, Tukey's HSD). A notable observation is that the context CS did not appear to induce continual *Arc* expression over the entire 30 minutes, but rather appeared to induce *Arc* during the initial 5–10 minutes of context exploration. This is evident by the fact that control animals showed a robust cytoplasmic *Arc* signal with minimal nuclear signal. The “punctuate” nature of the response to context made it possible to assess convergence because it was clear that nuclear *Arc* in the DS group was provoked by shock and not by context. Two groups display a nuclear *Arc* signal which can be clearly identified as responsive to shock-US delivery: the DS and LI groups (group main effects: *F*_3,12_ = 37.40, *P*<.001; *P*'s<.01 for DS/LI vs. control, Tukey's *HSD*). Interestingly, a significantly larger number of US-responsive cells were seen in the DS group than in the LI group (*P* = .01, Tukey's *HSD*), suggesting that exposure to a novel CS context potentiates the US response in BLA [22].

DS exposure, the only condition which supported robust one-trial fear conditioning, resulted in a significant number of neurons displaying both cytoplasmic and nuclear expression of *Arc* mRNA (group main effects: *F*_3,12_ = 33.08, *P*<.001; *P*'s<.001 for DS vs. all other groups, Tukey's *HSD*) (Figure 2D). The total number of neurons sampled in BLA can be examined in Table 1, which also includes the number and percentage of neurons displaying *Arc^+^* staining, as well as a breakdown of staining in only the cytoplasmic and/or nuclear compartments. The total percentage of cells that displayed *Arc* ^+^ staining did not differ between the DS group (18.3%), which learned, and the IS and control groups (∼16%), which did not. However, the percentage of cells that showed dual *Arc* ^+^ activation (in both the nuclear and cytoplasmic compartment) clearly distinguished the group that learned (DS = 3.9%, *P*<.001) from all other groups (0.6–1.3%).

**Table 1:** Number and Percentage of *Arc*+ neurons in sampled area of BLA.

				# neurons that showed *Arc+* staining in:		
Group	n	Total # of Neurons	Total # of *Arc+* Neurons	Cytoplasm only	Nucleus only	Both Nuc. & Cyto.
Control	4	323±7.15	50.3±10.2	40.8±9.9	5.25±1.0	4.25±0.6
			(15.6%)	(13.9%)	(3.0%)	(1.3%)
IS	4	338±9.10	56.3±8.90	49.5±9.7	1.71±.85	3.5±0.6
			(16.5%)	(15.5%)	(2.0%)	(1.0%)
DS	4	330±7.37	60.8±4.02	25.3±3.6	23.5±2.9	13±1.3**
			(18.3%)	(11.3%)	(11.0%)	(3.9%)
LI	4	326±11.6	29.3±1.54	5.00±.91	22.3±2.7	2.0±0.6
			(9.0%)	(2.1%)	(7.4%)	(0.6%)

** *P*<.001.

It is also noteworthy that the number of neurons showing *only* nuclear staining did not differ between DS and LI groups (Table 1), indicating that the observed enhancement in US-responding for the DS group was largely attributable to the presence of dual activated neurons.

### *A*rc catFISH in the hippocampus

**Figure 3:**
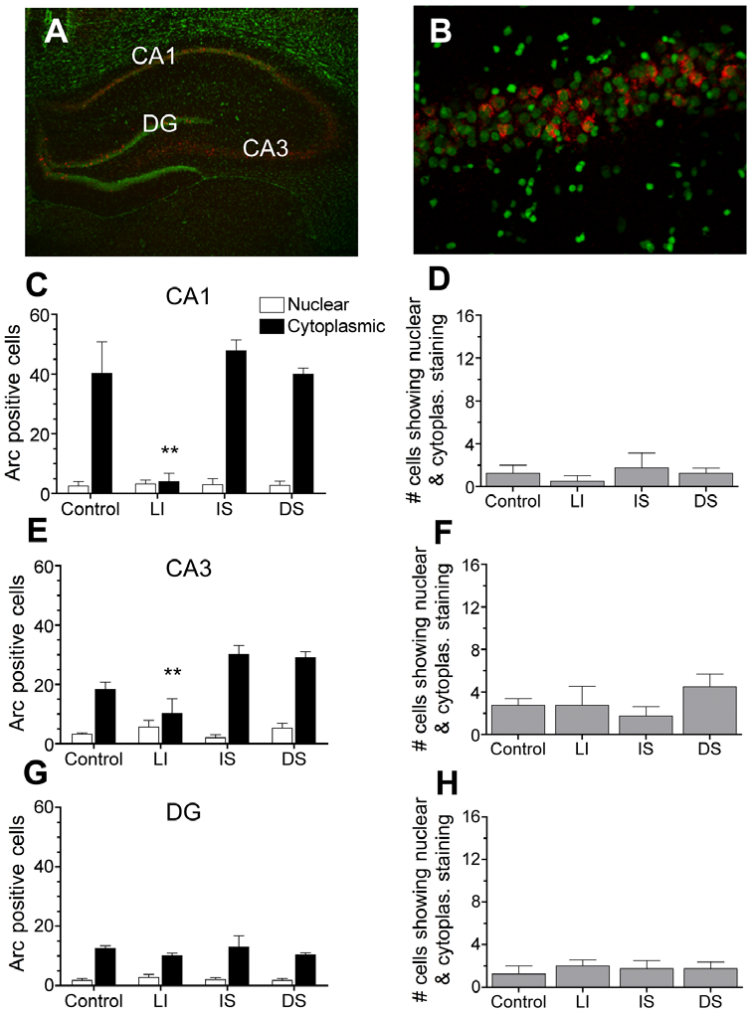
*Arc* induction in the hippocampus is sensitive to the CS, but not the US. (A) A representative image (4×) of the CA1, CA3, and DG regions of hippocampus displaying *Arc^+^* staining following DS fear conditioning. (B) A higher magnification (40×) image of CA1 showing primarily cytoplasmic *Arc* ^+^ staining. (C) Total number of cells in CA1 showing *Arc^+^* cytoplasmic and nuclear staining across groups (n = 4 rats/group). Robust cytoplasmic staining is seen following novel context CS exposure (as compared to LI group, **P<.001), but little nuclear staining is seen following shock. (D) No evidence for CS-US convergence could be found in CA1. (E–F) CA3 showed similar patterns of *Arc* ^+^ staining to CA1 with DS and IS animals showing significantly higher cytoplasmic (as compared to LI group, **P<.01). (G–H) DG showed relatively little *Arc* ^+^ staining that did not differ between groups. Data are represented as means±SEM.

In hippocampus, introduction to the novel context CS was effective in promoting *Arc* expression, whereas exposure to a familiar context CS and/or delivery of shock US were not (Figure 3A–B). Specifically, control, DS and IS animals showed comparable cytoplasmic *Arc^+^* staining in CA1 that was significantly greater than LI animals (Figure 3C) (group main effects: *F*_3,12_ = 11.27; *P* = .001; *P's<*.005 Tukey's *HSD*). In CA3, higher levels of cytoplasmic *Arc* were again seen in IS and DS as compared to LI animals (group main effects: *F*_3,12_ = 7.41; *P* = .005; *P*'s<.01, Tukey's *HSD*), whereas control animals showed intermediate *Arc* that did not differ from other groups (Figure 3E). Staining in DG was relatively low and did not differ between groups (Figure 3G). Unlike the BLA, hippocampal regions from DS and LI groups did not show significant nuclear *Arc* staining to shock. Not surprisingly, dual activation of *Arc* in cytoplasmic and nuclear compartments was not detected in the hippocampal neurons of DS animals (Figure 3D,F,H).

### *N*-methyl-D-aspartate (NMDA) receptor blockade and *A*rc catFISH

Given that BLA showed evidence of CS/US convergence, and that this pattern emerged only when animals learned, we then investigated whether NMDA receptor blockade, which prevents fear conditioning, similarly alters evidence of convergence in this region. 5-methyl-10,11-dihydro-5H-dibenzo[a,d]cyclohepten-5,10imine (MK801; a noncompetitive NMDA receptor antagonist) was administered systemically (0.3 mg/kg, *i.p*.) [25], [26] just before animals began DS training. Systemic administration was necessary as targeted infusions would require the presence of cannulae, which would impede rapid extraction of the brain and could damage the BLA, thereby interfering with catFISH analysis.

**Figure 4:**
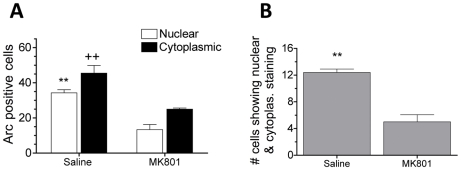
Treatment with MK801 prior to conditioning reduces neuronal convergence in BLA. (A) catFISH analysis showed that MK801-treated animals had significantly lower levels of CS-induced *Arc* (cytoplasmic staining, ++*P* = .01) US-induced *Arc* (nuclear staining, **P = .001) than saline animals. (B) Treatment with MK801 significantly reduced the number of neurons showing dual *Arc* ^+^ staining in both the cytoplasmic and nuclear compartments (**P = .001). Data are represented as means±SEM.

Behaviorally, treatment with MK801 effectively blocked one-trial contextual fear conditioning using our delayed protocol (Figure S1A–B). In animals used for catFISH analysis, MK801-DS rats vigorously reacted to footshock and exhibited impaired post-shock freezing (*t_7_* = 2.58, *P* = 0.036) (Figure S1C). Subsequent examination of *Arc* mRNA showed that introduction to the novel context CS and delivery of footshock US promoted robust *Arc* induction in BLA neurons of saline-DS animals (Figure 4A). In comparison, MK801-DS animals had significantly reduced *Arc^+^* staining in response to the CS and US (*t_7_* = 3.62, *P* = .01 for cytoplasmic staining; *t_7_* = 5.71, *P* = .001 for nuclear staining). The number of cells showing both nuclear and cytoplasmic *Arc* ^+^ staining was also significantly lower in MK801-DS animals compared to saline-DS animals (*t_7_* = 6.66, *P*<.001) (Figure 4B).

## Discussion

Fear conditioning is an adaptive, defensive associative learning task that can be acquired in a single trial [27]. Our behavioral results indicate that contextual fear conditioning can reliably occur in a single trial even when delivery of footshock is delayed for 26 min after introduction to a novel chamber (DS animals). Thus, the temporal parameters of contextual fear conditioning in the present studies conform to those needed for catFISH analysis. Specifically, they allowed us to determine whether cells in BLA and/or hippocampus are convergently activated by the CS and US, and whether convergence is specific to pairings that are effective in promoting fear learning (DS) as compared to those which are not (IS; LI). The location of such convergent cells was of particular interest as both BLA and hippocampus have been implicated in contextual fear conditioning [6]–[11], [21]–[23].

Previous contextual fear conditioning studies have shown that amygdalar neurons undergo learning-associated changes in neural activity [e.g., 9], but these plasticity-associated changes do not actually demonstrate that those neurons were responsive to both CS and US during learning. In the present studies, catFISH served as a functional imager that allowed us to visualize patterns of neuronal activation in response to the CS and US in animals that were awake and acquiring a one-trial learned association. The analysis demonstrates that during acquisition of contextual fear conditioning CS and US information converges on a subset of cells in the BLA, but not in dorsal hippocampus, only when presentation of the stimuli are effective in promoting learning. In fact, our data provide no indication that dorsal hippocampus is responsive to a single footshock US. This pattern of results supports the contention that fear memories, including contextual fear memories, are encoded in the amygdala and that the role of dorsal hippocampus in these associations is to process and transmit contextual CS information, such as novelty [30]–[32], [35].

Context novelty is clearly critical to rapid learning under these conditions. While LI animals did show moderate levels of postshock freezing during conditioning, they showed no evidence of learning when tested 24 hours later. Furthermore, overall *Arc* expression in BLA was notably lower for the LI group than in all other groups (Table 1). This appears to be largely due to the lack of cytoplasmic *Arc^+^* staining that accompanies exposure to a novel (but not familiar) context. The LI group did show significant nuclear *Arc* in response to the shock, but this cellular response to the US was not as strong as in DS animals. Finally, since there was minimal response to the familiar context CS, little to no convergence was seen in LI animals. This lack of convergence parallels the lack of fear conditioning in this group.

To assess whether activation of NMDA receptors is necessary for convergent *Arc* ^+^ activation and one-trial context fear learning in our paradigm, we tested the effects of the NMDA receptor antagonist, MK801, on both learning and *Arc* induction after conditioning. Consistent with previous reports that NMDA receptors play a crucial role in fear conditioning [36]–[38], we found that systemic injection of MK801 effectively blocked contextual fear conditioning. Paralleling the behavioral effect, MK801 significantly altered patterns of *Arc* expression. *Arc* ^+^ staining in response to the CS and US was reduced in BLA neurons and there was little evidence of dual activation.

The low percentage of cells in BLA showing dual activation (3.9%) implies that, during a single learning trial, convergently activated neurons are quite sparsely distributed. This could reflect the fact that the BLA, in addition to fear conditioning, is also involved in appetitive, sexual and several other motivated behaviours [39]. Regardless, it is important to note that, unlike the total number of *Arc* ^+^ cells, the appearance of convergently activated neurons in BLA is specific to conditions which support learning. Table 2 offers an overview of the relationship between conditions that are effective or ineffective in promoting learning and the pattern of *Arc* induction in cells of the amygdala and hippocampus. The appearance of significant levels of dual activation in amygdala clearly characterizes conditions supportive of learning and not conditions which are not.

**Table 2:** Relative levels of nuclear and cytoplasmic *Arc* in the BLA and Hippocampus of various groups that received CS and/or US exposure.

	BLA			Hippocampus (CA1 & CA3)			
Groups	Cytoplasmic	Nuclear	Both	Cytoplasmic	Nuclear	Both	Learned CS-US Association?
Exp. 1							
Control	++	-	-	+	-	-	no
IS	++	-	-	++	-	-	no
DS	++	++	+	++	-	-	yes
LI	-	+	-	-	-	-	no
Exp. 2							
DS-Saline	++	++	+	na	na	na	yes
DS-MK801	+	+	-	na	na	na	no

na, not applicable.

One subtle feature of the *Arc* response to the US deserves mention as it may bear a critical relationship to the appearance of dual activation. The number of neurons in BLA that are *Arc* ^+^ after footshock is significantly larger when it occurs in a novel (rather than a familiar) context CS, and only the novel CS yields one-trial learning and convergent CS-US activation. One potential explanation of the elevation in US responding could be an alteration in excitability of a subset of neurons in BLA that receive strong CS input and weak US input such that, when a novel CS precedes the US, these neurons become more sensitive to subsequent US input and convergent activation is seen. Interestingly, the increase in the number of activated neurons to the US corresponds to the number of neurons that display dual activation (Table 1), suggesting a critical link. Although the notion of US input as weak runs counter to prevailing views of the nature of associative learning (as well as the associative or Hebbian LTP model of fear conditioning [36], [37]), we postulate that the ‘weakness’ of US input may be limited to this particular subset of neurons. It is our view that the potentiation of US responding by prior exposure to a novel CS is not a coincidence unique to this set of data. An identical pattern emerged recently in catFISH analysis of CS-US convergence during conditioned taste aversion (CTA) learning [33]. More *Arc* ^+^ cells were seen in response to the US when it followed a novel CS taste than when it occurred alone, after a familiar taste or when it preceded a novel taste (backward conditioning). The consistency of this pattern and its association with both effective learning and convergently activated cells argues for an important role for this potentiation in excitability. Enhancement of neural response systems by recent exposure to novel stimuli has been reported to occur in hippocampus, where exposure to a novel context can enhance induction and maintenance of long-term potentiation (LTP) as well as long-term memory for an avoidance task [34], [35]. Our evidence supports a similar process in the amygdala during fear conditioning and taste aversion learning.

In conclusion, the results from the present studies support the view that the amygdala is a critical locus of fear conditioning. Our *Arc* data not only complement earlier electrophysiological studies, which showed sustained fear learning-associated increases in neural activities and LTP [13]–[15], and cellular-molecular studies, which revealed that *Arc*/*Arg*3.1 immediate early gene activation is a crucial component of the molecular cascade underlying fear conditioning [16]–[18], [40], but also provide crucial visual evidence that a population of BLA neurons receives convergent information at the time of training and does so only when the CS-US arrangement produces fear conditioning.

## Methods

### Subjects

Experimentally naïve adult male Sprague Dawley rats (initially weighing 250–275 g) were individually housed and maintained on a reverse 12 h light/dark cycle (lights on at 7:00 PM) with *ad libitum* access to food and water. All experiments were conducted during the dark phase of the cycle and in accordance with guidelines approved by the Institutional Animal Care and Use Committee at the University of Washington.

### Behavioral Procedures

Contextual fear conditioning used a modular operant test chamber (27 cm width×28 cm length×30.5 cm height; Coulbourn Instruments, Whitehall, PA) located in an acoustic isolation room. The grid floor was composed of 16 stainless steel bars (4.5 mm diameter) spaced 17.5 mm center to center and wired to a Coulbourn precision-regulated animal shocker. Floor grid and base pan were washed thoroughly with tap water and completely dried before conditioning and testing.

On conditioning day all animals were placed in an experimental chamber (wiped with 5% ammonium hydroxide solution) where they remained for 30 min after which they were returned to their home cage. Control rats received chamber exposure alone; IS (immediate shock) rats received footshock (2 mA, 5-sec) immediately upon being placed in the chamber; DS (delayed shock) rats received footshock 25 min and 55 sec after introduction to the chamber; and LI (latent inhibition–delayed shock) rats were treated exactly like DS rats except that they were pre-exposed to the chamber for 10 consecutive days (30 min each day) prior to conditioning. Post-shock freezing was measured for 4 min. The next day rats were placed back in the trained context for 8 min of context testing. Freezing data were collected via a 24 cell infrared activity monitor mounted on top of the chamber and connected to the Coulbourn Instruments LabLinc Habitest Universal Linc System [41], [42].

Separate animals were used for catFISH analysis and were conditioned as described above. Postshock freezing was measured for 4 min after which animals were sacrificed by guillotine (30 min after introduction into the context).

### Fluorescent *in situ* hybridization (FISH)

Brains were rapidly extracted, fresh frozen, and stored at −80°C. Forebrain tissue was sectioned into 20 µm coronal slices using a cryostat and mounted onto slides. Regions containing BLA and the CA1, CA3 and dentate gyrus of the hippocampus approximately –3.2 mm from bregma were selected for in situ hybridization. Digoxigenin-labeled *Arc* riboprobes were generated from a modified cDNA plasmid (provided by Paul Worley) and flourescent in situ hybridization and analysis were carried out as described elsewhere [19], [20], [33]. In brief, *Arc* signal was visualized using the Cyanine 3 TSA system (Perkin Elmer); nuclei were counterstained with Sytox Green (Invitrogen).

### Confocal microscopy and catFISH analysis

One section corresponding to each of the above regions was analyzed per rat (Figure S2). The compartmental analysis of *Arc* staining was done blind using Metamorph computer software following image capture on a Leica SL microscope (20× objective lens, 1-µm- thick optical sections) using GrHe/Ne and Argon lasers. Z-series stacks were constructed and analyzed on the MetaMorph 6.0 program as previously described [33]. Briefly, neurons were scored as positive for cytoplasmic staining if a ‘halo’ of signal was found to be encircling at least 75% of the nucleus, and were scored as positive for nuclear staining if robust foci with high saturation was found within the confines of the nucleus.

### Statistics

Analyses of behavioral data were performed using one-way ANOVA followed by Tukey's honestly significant difference (HSD) *post hoc* test. For experiments with only two groups, independent t-test analysis was used. Analysis of *Arc* positive neurons was also carried out with one-way ANOVA (4 groups) or independent samples t-test (2 groups) on SPSS (v. 15.0) software.

## Supporting Information

Figure S1 Treatment with MK801 prior to conditioning reduces post-shock freezing and abolishes learning. (A) Freezing behavior during training. Both saline-treated and MK801-treated groups reacted vigorously to footshock US presentation, but only saline animals showed reliable postshock freezing (average freezing over 4 min post shock period indicated by grey-filled symbols, P = .033). (B) When tested 24 hours after conditioning, saline-treated animals showed significantly greater freezing to the context than animals treated with MK801 (P = .011). (C) Animals used for catFISH analysis showed similar patterns of postshock freezing as behaviorally test animals. Once again, saline-treated animals showed significantly greater postshock freezing (grey-filled symbols) compared to MK801-treated animals (P = .036).
(5.28 MB TIF)

Figure S2 Representative images of brain sections analyzed.(A) A schematic drawing of a coronal slice at −3.12 mm from bregma (modified from Paxinos and Watson, 1997). (B) A representative micrograph of a 20 µm slice containing sampled regions of BLA and CA1, CA3, and DG subregions of dorsal hippocampus. All whole neurons within the demarcated regions were scored for Arc signal.
(4.50 MB TIF)
