# Supplementary figures and images for: Functional Imaging of Stimulus Convergence in Amygdalar Neurons during Pavlovian Fear Conditioning
Source: PLoS One. 2009 Jul 7;4(7):e6156. doi: 10.1371/journal.pone.0006156 (PMC2701998; doi:10.1371/journal.pone.0006156)

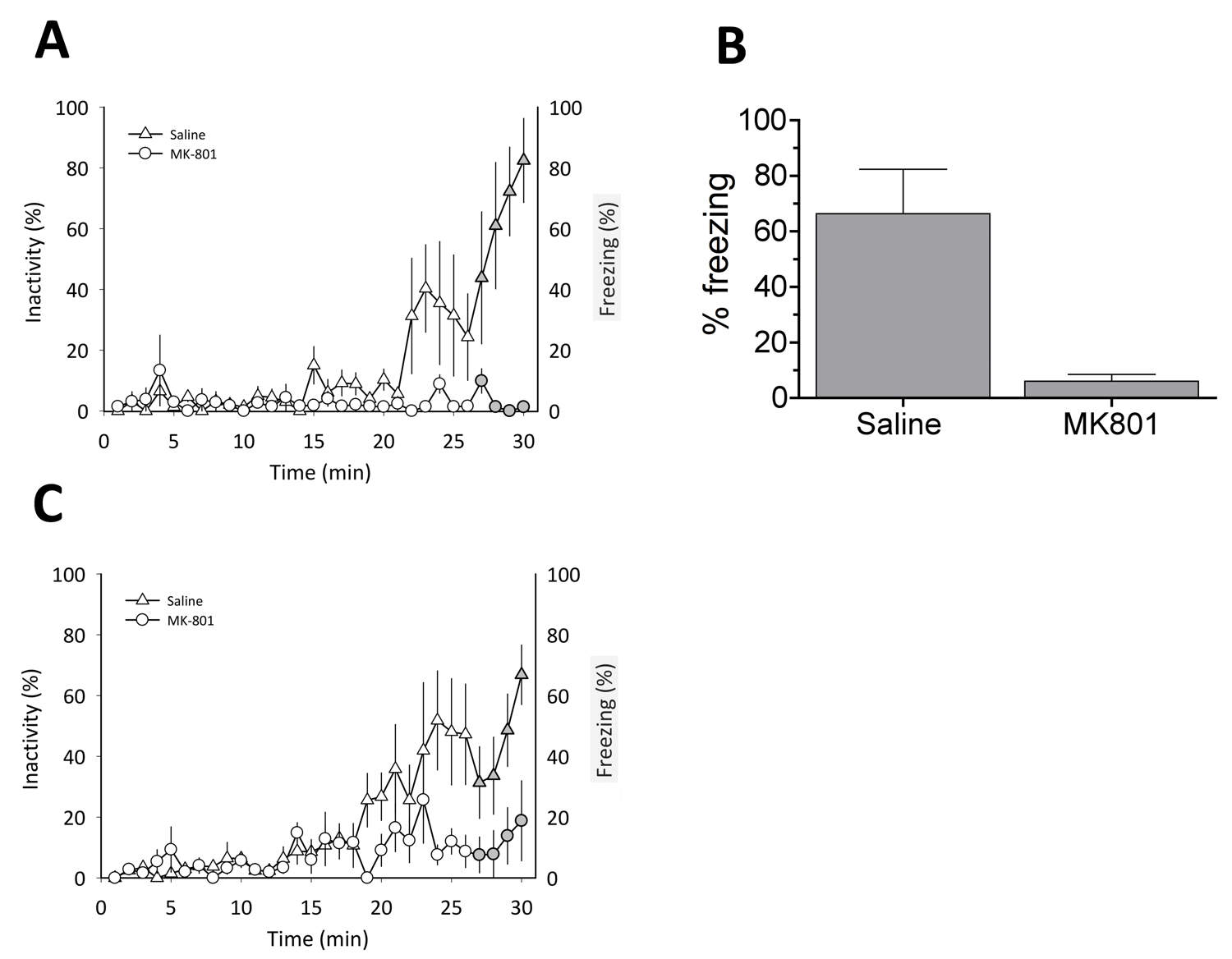

Supplement: Figure S1 — Treatment with MK801 prior to conditioning reduces post-shock freezing and abolishes learning. (A) Freezing behavior during training. Both saline-treated and MK801-treated groups reacted vigorously to footshock US presentation, but only saline animals showed reliable postshock freezing (average freezing over 4 min post shock period indicated by grey-filled symbols, P = .033). (B) When tested 24 hours after conditioning, saline-treated animals showed significantly greater freezing to the context than animals treated with MK801 (P = .011). (C) Animals used for catFISH analysis showed similar patterns of postshock freezing as behaviorally test animals. Once again, saline-treated animals showed significantly greater postshock freezing (grey-filled symbols) compared to MK801-treated animals (P = .036). (5.28 MB TIF) [file pone.0006156.s001.tif]

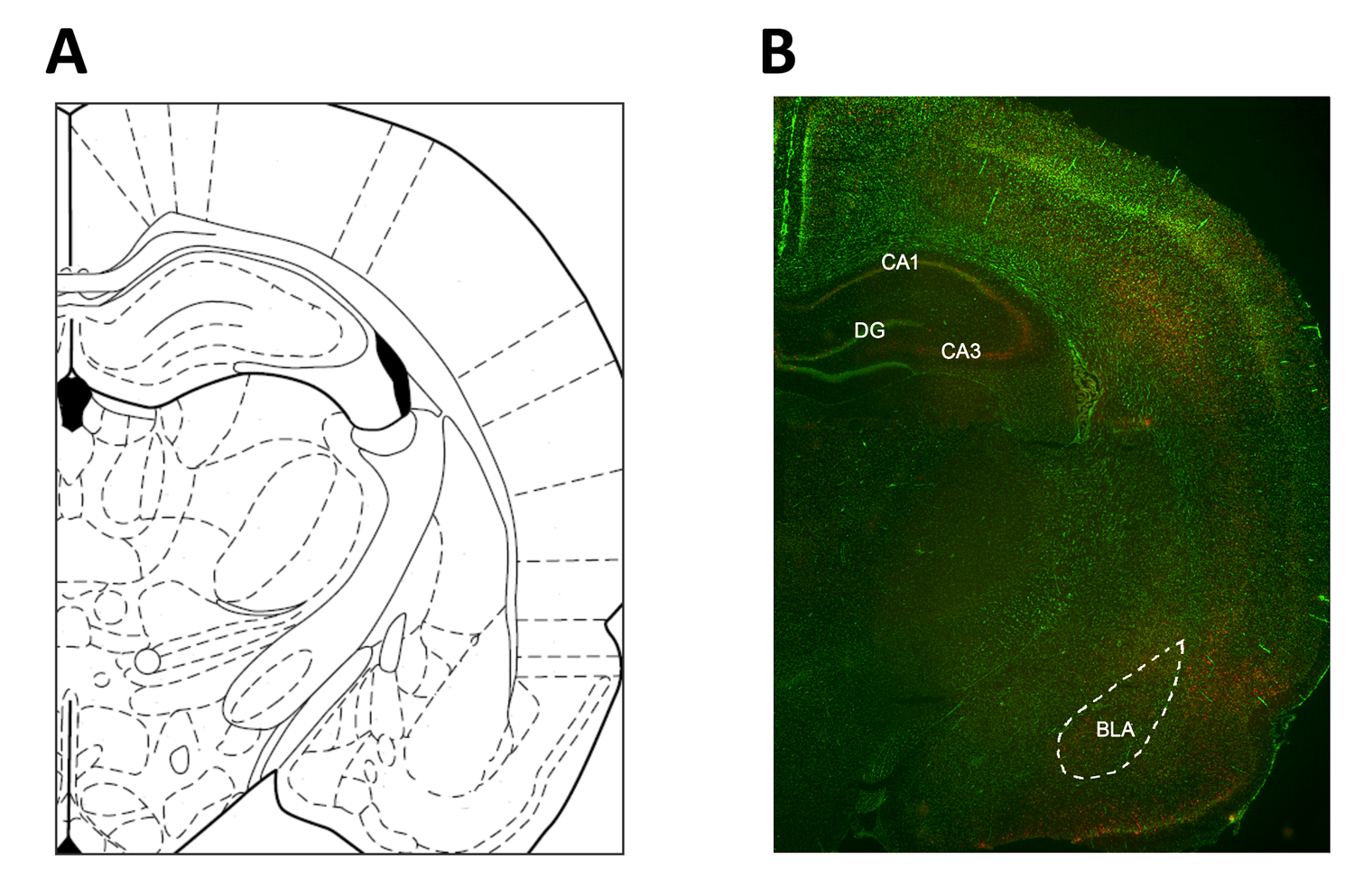

Supplement: Figure S2 — Representative images of brain sections analyzed.(A) A schematic drawing of a coronal slice at −3.12 mm from bregma (modified from Paxinos and Watson, 1997). (B) A representative micrograph of a 20 µm slice containing sampled regions of BLA and CA1, CA3, and DG subregions of dorsal hippocampus. All whole neurons within the demarcated regions were scored for Arc signal. (4.50 MB TIF) [file pone.0006156.s002.tif]
